# Case Report: Upper airway obstruction due to rheumatoid arthritis

**DOI:** 10.12688/f1000research.22256.1

**Published:** 2020-02-18

**Authors:** Mohan Rudrappa, Laxmi Kokatnur, Sanket Shah

**Affiliations:** 1 Department of Medicine, Mercy Hospital, Joplin, MO, USA

**Keywords:** Rheumatoid arthritis, Upper airway obstruction, cricoarytenoid joint, Odontoid fracture

## Abstract

Rheumatoid arthritis (RA) is a common autoimmune disease characterized by inflammation of small joints. Small synovial joints in the larynx can also become affected, and laryngeal involvement is seen in more than half of patients with RA. As most patients have subtle symptoms and indolent course, they are either misdiagnosed or undiagnosed. The acute worsening of cricoarytenoid arthritis can cause sudden upper airway obstruction and may require emergency intubation or tracheostomy. This life-threatening condition is described in only a handful of cases in the medical literature. Physicians should be aware of this rare but life-threatening consequence of RA. We present a case of sudden and severe upper airway obstruction secondary to laryngeal involvement in a patient with long-standing RA.

## Introduction

Rheumatoid arthritis (RA) is a common autoimmune disease that affects 1% of the adult population ^1^. It is characterized by the development of both articular and extra-articular lesions with a predilection for small joints. Laryngeal involvement in patients with RA is invariably underdiagnosed due to its subtle clinical features, but at times it can present as a life-threatening emergency. The involvement of the cricoarytenoid joint can lead to paralysis of the vocal cord leading to upper airway obstruction. We present a case of sudden and severe upper airway obstruction secondary to laryngeal involvement in a patient with long-standing RA.

## Case presentation

A 75-year-old woman with a history of coronary artery disease (status post- coronary artery bypass graft seven years ago) and RA (diagnosed ten years back being treated with 10 mg/day of prednisone and 200 mg/day of Plaquenil) was admitted to the hospital for evaluation of new-onset seizure. She was followed by a rheumatologist when she was living in Texas several years back, and presently it is managed by her primary care physician. The patient has tried several medications for her RA, including Imuran and monthly injections, and reports that only prednisone helps her arthritis.

**Figure 1.**
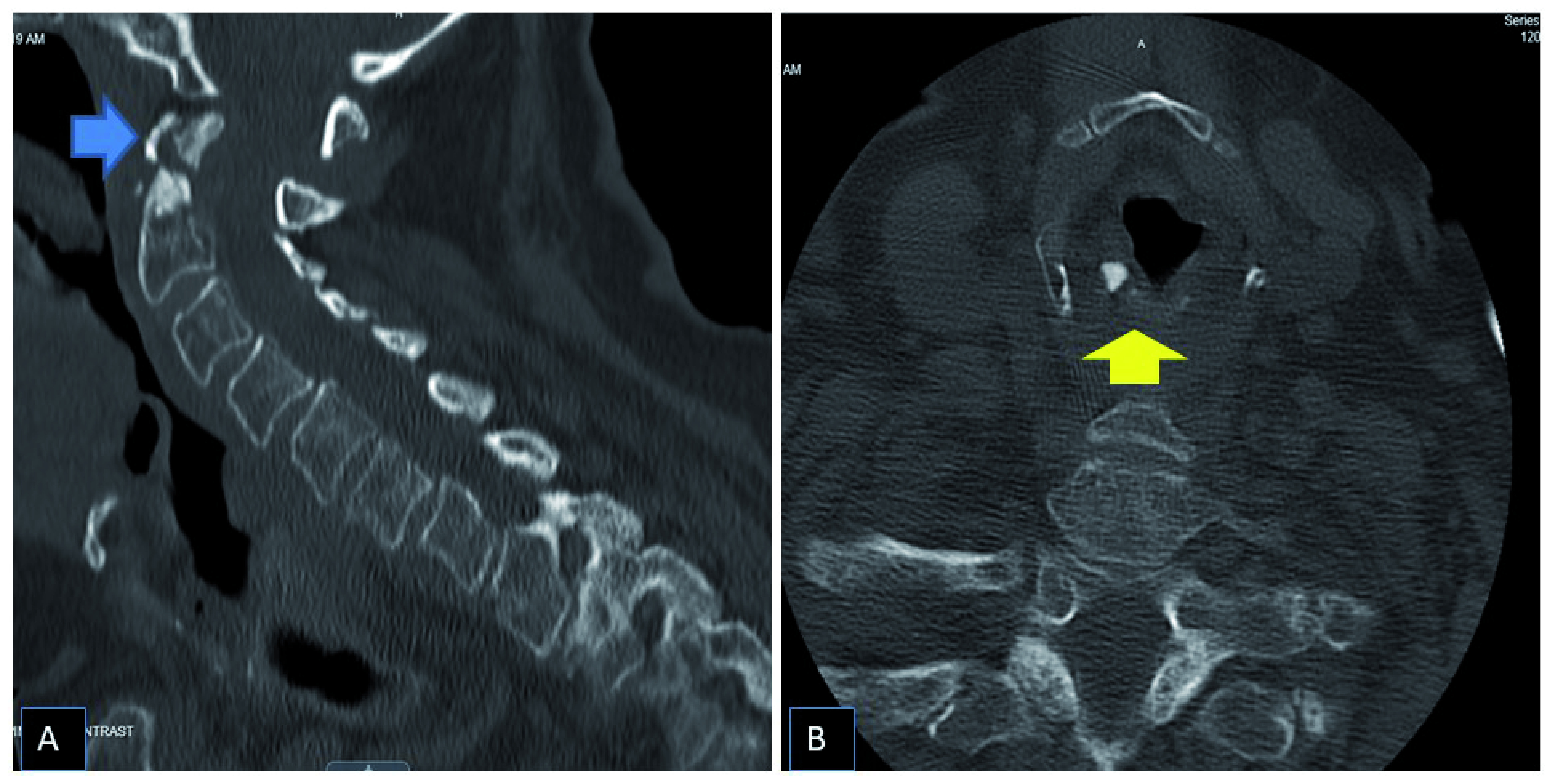
CT scan of the cervical neck showing Type II odontoid fracture (blue arrow) and sclerosis of the arytenoid cartilage (yellow arrow).

A CT scan of the head done in the Emergency Room did not show any acute intracranial process but suggested the possibility of a cervical spine fracture. Dedicated CT of the cervical spine did confirm type II odontoid fracture ( Figure 1A). The patient was evaluated by Neurosurgery and opted for conservative management with a cervical collar as the patient did not have any neurological symptoms. On the night of admission to the medical floor, the patient developed hypoxemia and was started on supplemental oxygen through the nasal cannula. However, her saturation continued to deteriorate, leading to the development of severe bradycardia was transferred to the Intensive Care Unit for further management. Urgent CT pulmonary angiogram did not show any embolism nor pleuro-parenchymal changes. In the intensive care unit, the patient was initially treated with nasal cannula oxygen and later high flow oxygen without any improvement in saturation. The patient reported some breathlessness but was not in distress. Clinical examination was normal. This newly diagnosed unexplained hypoxemia was attributed to the hard cervical collar leading to airway compromise. However, despite trying a different sized hard collar and soft cervical collar, she continued to show hypoxemia. The patient was later started on with Bilevel Positive Airway Pressure (BIPAP) with improvement in saturation. By evening, the saturation dropped to 70% despite the up-titration of BiPAP settings and developed sinus bradycardia with a heart rate of 30 beats/min. The patient was emergently intubated. Due to cervical cord fracture, intubation was done with a fibro-optic pediatric bronchoscope, and seven number endotracheal tube was placed without any difficulty. Arytenoid swelling was noted during intubation, but due to emergency procedure and lack of video monitoring, it was not explored further. 

**Figure 2.**
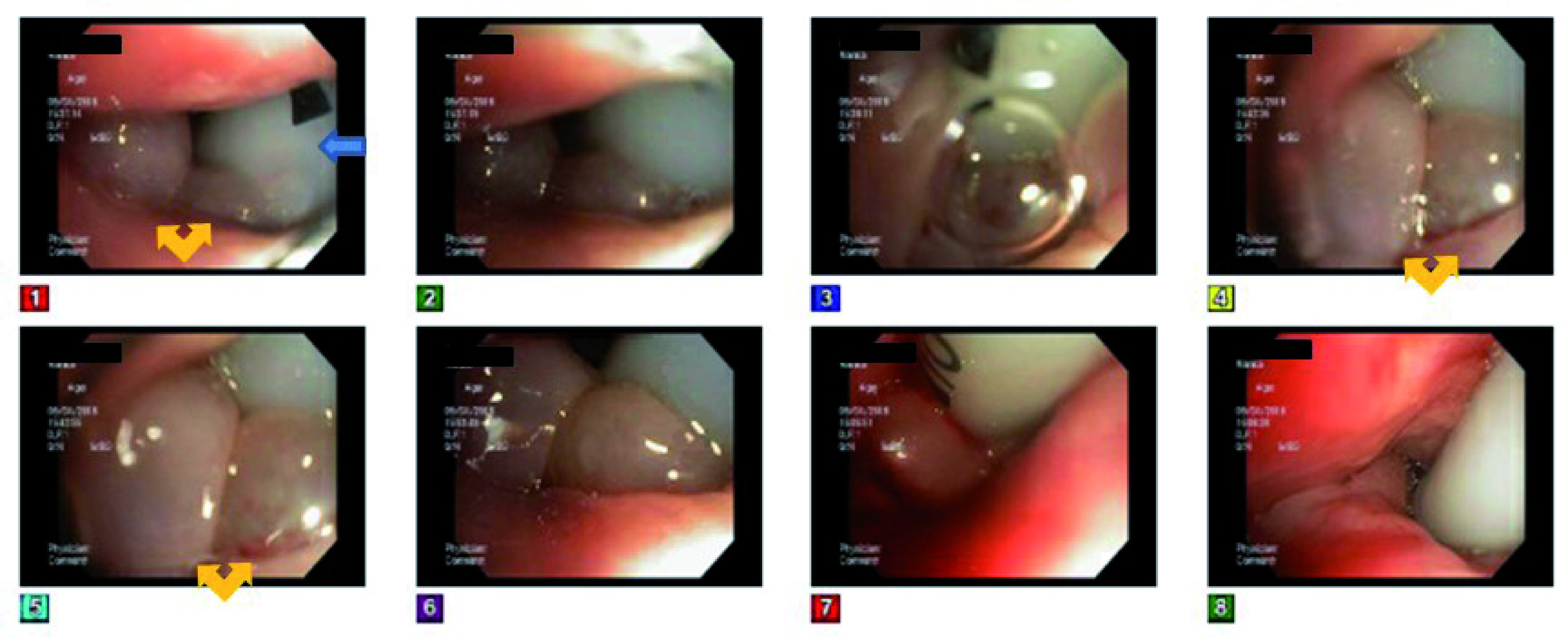
Video bronchoscopy images showing enlarged arytenoid appendages (yellow arrows). Endotracheal tube (blue arrow) can be seen entering the vocal cords.

The next day, video bronchoscopy performed through the endotracheal tube did not reveal any endobronchial pathology. However, bronchoscopy through the right nostril showed evidence of significant arytenoid swelling compromising the upper airway space ( Figure 2). On reviewing the CT scan of the cervical spine done in the emergency room, there was evidence of arytenoid cartilage sclerosis and cricoarytenoid joint involvement secondary to RA ( Figure 1B). Further collateral history was obtained from the patient's daughter, who denied any prior history of speech, swallow, or breathing problem. The patient has only multiple joint pain, stiffness, and deformity without any other organ involvement RA. The patient smoked a pack of cigarettes several years back, quit smoking seven years back when she had heart surgery. She has not been diagnosed with chronic obstructive pulmonary disease nor with any sleep-related problems before.

Based on these findings, the patient was diagnosed with sudden onset of cricoarytenoid arthritis due to RA. After intubation, the patient did not show any evidence of desaturation but developed ventilator-associated pneumonia and was treated with antibiotics. The patient was treated with 60 mg/day prednisone for a week, along with antibiotics and bronchodilators. She did not show any improvement in upper airway obstruction without any air leak around the vocal cords during the air-leak test done on the ventilator. The patient underwent tracheostomy and was transferred to Long-Term Acute Care for further management after a week of hospitalization. She was later weaned from the ventilator but could not be decannulated from tracheostomy. She was discharged with 6 mm Shiley™ tracheostomy tube along with supplemental oxygen. During her follow-up visit to ENT clinic, a direct laryngoscope continued to show arytenoid swelling and the vocal cords fixed in the midline position. The patient wase also followed up with rheumatology and stared on Imuran therapy and being worked up for biological agents. Steroids were taperedd off and stopped completely.

## Discussion

Laryngeal involvement in patients with RA seen in 31–75% of patients and histological laryngeal involvement in postmortem studies can be seen in up to 90% of patients ^2, 3^. It can present as mild mucosal edema with inflammation, epiglottitis, cricoarytenoid arthritis, and rheumatoid vocal fold nodules. Cricoarytenoid joint involvement leads to significant edema and redness of the arytenoid cartilage, inter arytenoid pachydermia, impaired mobility, or fixation of the arytenoid or vocal cords ^4^. Bilateral involvement of the cricoarytenoid joint is only seen in 13–30% of patients with RA ^2^. Chronic inflammatory changes in the joints can result in ankylosis and decreased mobility of one or both vocal cords. Although the severity of the RA is a risk factor of laryngeal involvement, the severity of laryngeal involvement does not correlate with the disease severity ^5^.

Clinical presentation depends on unilateral or bilateral involvement and the position of the vocal cords. Mild voice change, sore throat, foreign body sensation, throat swelling, aspiration, and dyspnea with stridor can be seen ^6^. For chronic symptomatic, e.g. upper airway disease, systemic intra-articular steroids have some symptomatic benefit. Surgical management, including tracheostomy, adenoidectomy, or arytenoidopexy, may be necessary if progressive airway obstruction occurs despite medical management. Some patients may present with sudden upper airway obstruction due to acute decompensation of chronic joint inflammation superimposed by laryngeal edema due to chronically immobile vocal cords, often triggered by an infectious process ^5^. The use of a laryngeal mask airway has been reported to exacerbate laryngeal swelling ^6^. In mild cases, the use of local anesthesia and intra-articular corticosteroids may be beneficial. If intubation is required, bronchoscopic intubation with a small lumen endobronchial tube is recommended. The involvement of the cervical spine and RA requires careful attention in neck manipulation during intubation ^6^.

Our case was unique in several ways compared to other cases described in the literature ^2, 3, 5, 6^. Our patient also had atraumatic type II odontoid fracture due to RA. The prevalence of radiological cervical spine involvement in RA is seen in 20–85% of patients, and the most prevalent abnormality is atlantoaxial subluxation, followed by impaction ^7^. Odontoid fracture is rare and only described in a few cases in the literature ^7– 10^. Prolonged steroid use can precipitate osteoporosis in the cervical spine and predispose to atraumatic fracture, as seen in our case. Our patient did not have any neurological symptoms, and hence, was treated with stabilization of the cervical spine with a hard collar. The new onset hypoxemia was initially attributed to the use of a hard collar and also posed challenges to emergency intubation. Our patient also had significant bradycardia during episodes of hypoxemia.

Treatment of our case also has a limitation. Bronchoscopy was not performed after tracheostomy to look for the vocal card position. The patient developed ventilator-associated pneumonia and was requiring 50% Fio2; hence this was deferred.

## Conclusion

Laryngeal involvement and atlantoaxial subluxation of the cervical spine are common and well described in patients with RA. Acute cricoarytenoid arthritis leading to sudden upper airway obstruction is rare. Also, an atraumatic odontoid fracture complication due to RA very rare. Both these rare complications occurring together have not, to the best of our knowledge, been described in the medical literature. Physicians taking care of patients with RA should be aware of these rare complications, which can be life-threatening.

## Consent

Written informed consent for the publication of the article, and any associated images was obtained from the patient.

## Data availability

All data underlying the results are available as part of the article and no additional source data are required.
