# Supplementary material for: Case Report: Upper airway obstruction due to rheumatoid arthritis
Source: F1000Res. 2020 Feb 18;9:119. [Version 1] doi: 10.12688/f1000research.22256.1 (PMC8383128; doi:10.12688/f1000research.22256.1)
Supplement: Peer review history

# Peer review: Reviewer response for version 1

**DOI:** 10.5256/f1000research.24550.r88077

**Published:** 2021-08-23

**Authors:** Manish Gupta

**Affiliations:** 1 Department of ENT, Maharishi Markandeshwar Institute of Medical Sciences & Research, Ambala, Haryana, India

- The case report has spelling errors in the last line of the Case Presentation section.
- The case doesn't talk about the role of EMG to differentiate between vocal cord paralysis and cricoarytenoid joint fixation.
- Trauma to the neck should be ruled out. It may present with supraglottic edema and a fractured spine.
- Episodes of seizure and possible cause are not explained.

Are enough details provided of any physical examination and diagnostic tests, treatment given and outcomes?

No

Is the case presented with sufficient detail to be useful for other practitioners?

Yes

Is sufficient discussion included of the importance of the findings and their relevance to future understanding of disease processes, diagnosis or treatment?

Yes

Is the background of the case’s history and progression described in sufficient detail?

Yes

Reviewer Expertise:

Otology, Rhinology, Laryngology, Head Neck Disease

I confirm that I have read this submission and believe that I have an appropriate level of expertise to confirm that it is of an acceptable scientific standard.

# Peer review: Reviewer response for version 1

**DOI:** 10.5256/f1000research.24550.r68830

**Published:** 2020-08-06

**Authors:** Waiz Wasey

**Affiliations:** 1 Family and Community Medicine, Southern Illinois University School of Medicine, Springfield, USA

The article brings to light a very important issue with RA involving the upper airway. Here the description of involvement of cricoarytenoid arthritis and swelling secondary to RA is aptly done. I had written a paper indicating how RA involvement of the cervical vertebrae led to worsening of Sleep apnea. Unfortunately my patient did not have an endoscopy done to see if there was any swelling involved as described here. The obstruction here was severe enough leading to tracheostomy tube placement, which indicates a severe obstruction possibility which primary care physicians, ER and Rheumatologists should be aware of. Although the incidence is very rare, such complications do happen. The paper also brings out an interest fact that RA inflicted patients are at increased risk of Ventilator associated pneumonia secondary to their immunocomprised state from the medications. 

 I concur that physicians taking care of patients with RA should be aware of any such complications that can arise and are potentially life threatening.

Are enough details provided of any physical examination and diagnostic tests, treatment given and outcomes?

Yes

Is the case presented with sufficient detail to be useful for other practitioners?

Yes

Is sufficient discussion included of the importance of the findings and their relevance to future understanding of disease processes, diagnosis or treatment?

Yes

Is the background of the case’s history and progression described in sufficient detail?

Yes

Reviewer Expertise:

Sleep Medicine

I confirm that I have read this submission and believe that I have an appropriate level of expertise to confirm that it is of an acceptable scientific standard.
